# Response Surface Optimized Ultrasonic-Assisted Extraction of Flavonoids from Sparganii Rhizoma and Evaluation of Their *in Vitro* Antioxidant Activities

**DOI:** 10.3390/molecules17066769

**Published:** 2012-06-04

**Authors:** Xinsheng Wang, Qinan Wu, Yanfang Wu, Guangyun Chen, Wei Yue, Qiaoli Liang

**Affiliations:** 1 Pharmaceutical School, Nanjing University of Chinese Medicine, Nanjing 210046, Jiangsu, China; 2 Chemical and Pharmaceutical School, Henan University of Science and Technology, Luoyang 471003, Henan, China; 3 Jiangsu Key Laboratory for TCM Formulae Research, Nanjing University of Chinese Medicine, Nanjing 210046, Jiangsu, China; 4 Pharmaceutical School, Xinxiang Medical University, Xinxiang 453003, Henan, China

**Keywords:** ultrasound-assisted extraction, response surface methodology, sparganii rhizoma, flavonoids, antioxidant

## Abstract

An efficient ultrasound-assisted extraction technique was employed to extract total flavonoids from Sparganii rhizoma. The optimum extraction conditions for the highest yield of total flavonoids were ethanol concentration 53.62%, ultrasonication time 29.41 min and ultrasound power 300 W, which were determined using response surface methodology. The extraction yields of the optimal ultrasound-assisted extraction were higher than using conventional extraction. The crude extract was then purified on a polyamide resin, whereby the flavonoids content in the purified extract increased to 94.62%. The antioxidant activities of the purified flavonoids including DPPH radical scavenging activity, ABTS^+^ radical scavenging activity, reducing power, hydroxyl radical scavenging activity and superoxide anion scavenging activity, were evaluated *in vitro*, which suggested that the flavonoids showed significant antioxidant activities. Rutin, kaempferol and formononetin were identified in the extract by comparing relative retention times and UV-Vis spectra with those of reference standards.

## 1. Introduction

Sparganii rhizoma (SR), the dried rhizome of *Sparganium stoloniferum* Buch.-Ham., is a traditional Chinese folk medicine that has long been used for the treatment of blood stasis and dysmenorrhea [1]. Pharmacological investigations have shown that flavonoids are one of the most important contributors to the biological activity of SR, including the anti-platelet and anti-thrombotic actions [2], analgesic and anti-inflammatory effect [3], inhibition of HeLa cell multiplication [4], anti-cancer activity [5]. These efficacies of the flavonoids have been associated with their antioxidant activities [6,7,8,9,10]. However, insufficient studies have been conducted on the role of flavonoids from SR on free radical management and antioxidant activity.

Different methods, including refluxing, boiling, heating and Soxhlet extraction, have been used for the extraction of flavonoids, however, the general disadvantages of all these methods are the potential losses of flavonoids due to oxidation, hydrolysis and ionization during extraction as well as the long extraction times [11]. Recently, various new extraction techniques have been developed for the extraction of the flavonoids from plants, including microwave-assisted extraction [12], ultrasound-assisted extraction [13], accelerated solvent extraction [14] and supercritical fluid extraction [15]. Among these, ultrasound-assisted extraction is an inexpensive, simple and efficient alternative to conventional extraction techniques.

In order to optimise the extraction conditions, including concentration of solvent, extraction time and ultrasonic power, response surface methodology (RSM) has been widely used. By establishing a mathematical model, RSM evaluates multiple parameters and their interactions using quantitative data, effectively optimising complex extraction procedures in a statistical way, thus reducing the number of experimental trials required [16]. Although RSM has been applied to optimise ultrasonic-assisted extraction of flavonoids in many studies [13,17], to the best of our knowledge, there are no reports yet about the application of RSM on ultrasonic-assisted extraction optimisation for the extraction of flavonoids from SR. In the present work, we used RSM to optimise the ultrasonic-assisted extraction of total flavonoids from SR. The antioxidant activities of flavonoids were evaluated using multi-test systems *in vitro*. The aim of our work was to establish the optimised parameters of ultrasound-assisted extraction for the flavonoids extract from SR and explore the potential antioxidant properties of the flavonoids, and offer scientific reference for development and utilization of the resource.

## 2. Results and Discussion

### 2.1. Optimization of Ultrasonic-Assisted Extraction Parameters for Flavonoids

#### 2.1.1. Fitting the Response Surface Model

Table 1 shows the yields of total flavonoids (*Y*) from SR obtained from all the experiments. Multiple linear regression was performed based on the results of Table 1 using the following quadratic polynomial model (Equation 1).


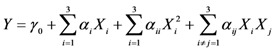
(1)


where *Y* is the predicted response, *γ_0_* is a constant, *α_i_,α_ii_* and *α_ij_* are the linear, quadratic and interactive coefficients of the model, respectively. Accordingly, *X_i_* and *X_j_* represent the levels of the independent variables, respectively. The response variable and the independent variables are related by the following second-order polynomial equation (Equation 2).


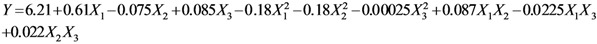
(2)

**Table 1:** Response surface Box-Behnken design (uncoded) and results for extraction yields of RS.

Run	*X_1_* (%, v/v)	*X_2_* (min)	*X_3_*(W)	*Y* (mg/g)
1	40	40	270	5.52
2	50	20	240	5.97
3	40	20	270	5.87
4	60	30	300	6.18
5	50	40	300	6.14
6	40	30	300	5.93
7	50	30	270	6.30
8	40	30	240	5.84
9	50	40	240	5.80
10	50	30	270	6.17
11	60	30	240	6.18
12	50	20	300	6.22
13	50	30	270	6.17
14	50	30	270	6.26
15	60	20	270	6.02
16	50	30	270	6.14
17	60	40	270	6.02

Table 2 presents the regression coefficients and the results of analysis of variance (ANOVA). It was clear that the model fit well with the response variables, because the model could explain the variability of most of the responses. The coefficient of multiple determination (*R*^2^) was 0.9213, suggesting that a very high correlation was achieved as the *R*^2^ value was higher than 0.8 [18]. Furthermore, the lack of fit test was used to verify the adequacy of the fit. In case of flavonoids, the lack of fit was not significant because it was at a 95% confidence level, as shown in Table 2. This indicates that the experimental data fit well with the model. Additionally, the *p* values were used as a tool to evaluate the significance of each coefficient, which in turn might indicate the pattern of the interactions between the variables. In this case, a smaller *p* value indicates a more significant corresponding coefficient. As shown in Table 3 the linear coefficients (*X_1_*, *X_2_* and *X_3_*) and quadratic term coefficients (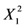,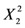) were significant, with a very small *p* value (*p* < 0.05), while the other term coefficients were not significant (*p* > 0.05).

**Table 2:** The regression coefficients and results of ANOVA.

Source	Coefficient	Sum of Squares	Df	Mean Square	*F* value	*P* value
Model		0.6043229	9	0.0671470	9.1037954	0.0041
γ *_0_*	6.20800					
*X_1_*	0.15500	0.1922000	1	0.1922000	26.0584931	0.0014
*X_2_*	−0.07500	0.0450000	1	0.0450000	6.1011040	0.0428
*X_3_*	0.08500	0.0578000	1	0.0578000	7.8365291	0.0265
*X_1_X_2_*	0.08750	0.0306250	1	0.0306250	4.1521402	0.0810
*X_1_X_3_*	−0.02250	0.0020250	1	0.0020250	0.2745497	0.6165
*X_2_X_3_*	0.02250	0.0020250	1	0.0020250	0.2745497	0.6165
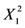	−0.17525	0.1293161	1	0.1293161	17.5326819	0.0041
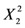	−0.17525	0.1293161	1	0.1293161	17.5326819	0.0041
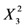	−0.00025	0.0000003	1	0.0000003	0.0000357	0.9954
*R^2^*	0.9213					
Residual		0.0516300	7	0.0073757		
Lack of Fit		0.0329500	3	0.0109833	2.3518915	0.2135
Pure Error		0.0186800	4	0.0046700		
Total		0.6559529	16			

#### 2.1.2. Analysis of the Response Surface

**Figure 1:**
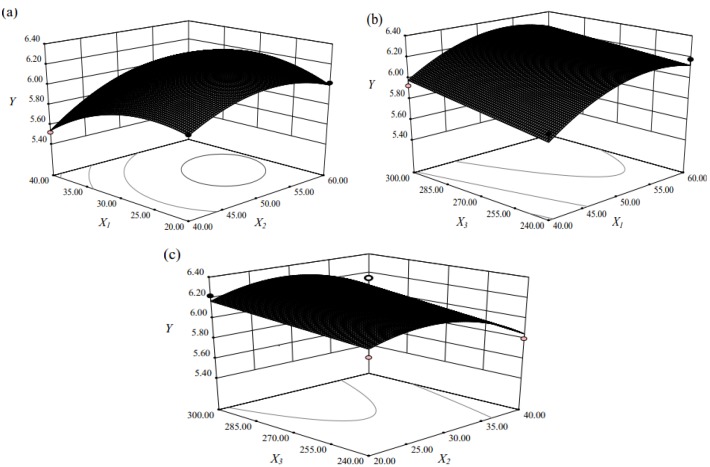
Response surface and contour plots for the effect of independent variables on extraction of the flavonoids: (**a**) ethanol concentration and extraction time; (**b**) ethanol concentration and ultrasound power; (**c**) extraction time and ultrasound power.

To provide a better visualization of the statistically significant factors derived from the statistical analysis, the response surface and contour plots for the effects of independent variables on the extraction of flavonoids is given in Figure 1. The plots showed effects of two factors on the response at one time while the third factor was kept at zero level in all figures.

Table 2 indicates that the total flavonoids yield and extraction parameters were quadratic with a good regression coefficient (*R*^2^ = 0.9213). As shown in Figure 1a, it can be concluded that the maximum total flavonoids extraction could be achieved when the ethanol concentration and extraction time were 53.62% and 29.41 min, respectively. The total flavonoids yield increased with an increase of ethanol concentration from 40.00% to 53.62% and then decreased when ethanol concentration was above 53.62%. The total flavonoids yield increased with prolonged extraction time from 20 to 29.41 min and then descended with extended extraction time. This observation was understandable because, on the one hand, ultrasound induces acoustic cavitation and the rupture of plant cells [19], both of which facilitate the penetration of the solvent into the plant cell to dissolve the objective constituents. A prolonged extraction time would allow all the plant cells to be completely cracked by acoustic cavitation, thus the extraction yield would increase within a certain time. On the other hand, completely ruptured plant cells would also allow various compounds such as insoluble and cytosolic substances to be released into the extraction solvent, thus limiting the solubility and permeability of the solvent [20]. In addition, the target constituents might also be re-adsorbed on the smashed plant particles, thus also affecting the yields of recovered compounds [21]. At this point, one would expect that increased ultrasonic power might increase the extraction yield. As shown in Figure 1b, increased extraction yield of total flavonoids was observed with increasing ultrasonic power from 240 W to 300 W. Obviously, ultrasound power was very important in improving the total flavonoids yield. The higher the ultrasound power was, the more solvent could enter cells and the more target compounds could permeate cell wall, which suggested that increased ultrasound power could enhance the total flavonoids yield. The result was similar to that previously reported by Zou *et al.* [22]. Figure 1c presents the interaction of extraction time and ultrasound power. It is obvious that maximum total flavonoids yield was achieved when extraction time was 29.40 min and ultrasound power was 300W.

#### 2.1.3. Verification of Predictive Model

According to the RSM test results, the optimal extraction parameters were as follows: ethanol concentration 53.62%, ultrasonic time 29.41 min and ultrasound power 300 W. To verify the suitability of the equation model used for predicting the optimum response values, the optimisation study was performed. The results are shown in Table 3 with the amounts of total flavonoids under the optimal conditions and solvent extraction conditions. No significant different (*p* > 0.05) was found between the experimental and predicted values of total flavonoids. Hence, the models can be used to optimise the process of total flavonoids extraction form SR.

**Table 3:** Predicted and experimental values of total flavonoids obtained under the optimal extraction conditions and solvent extraction (mg/g).

Extraction variables			Predicted value	Experimental value	Solvent extraction
*X_1_* (%)	*X_2_* (min)	*X_3_* (W)			
53.62	29.41	300	6.32	6.38 ± 0.13 ^a^	4.08 ± 0.22 ^a^

^a^ Mean ± standard deviation (n = 5).

#### 2.1.4. Flavonoid Compounds Identification

**Figure 2:**
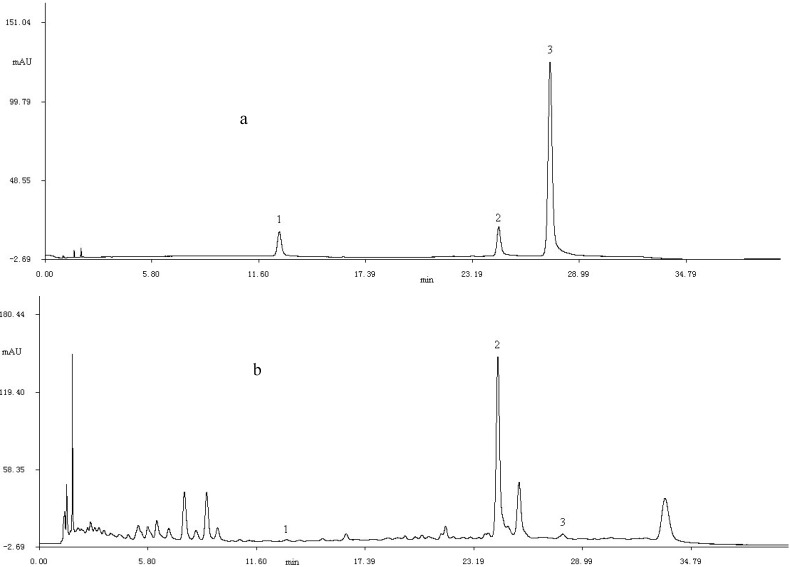
The HPLC chromatograms of standard samples (**a**) and purified flavonid (**b**) at 280 nm; 1. rutin; 2. kaemperol; 3. formononetin.

Figure 2 shows the HPLC chromatogram of standards and extract. By comparing relative retention time and UV-Vis spectra with those of reference standards, three flavonoid compounds, including rutin, kaempferol and formononetin, were indentified. It can be observed that the kaempferol is the predominant flavonoid.

### 2.2. *In Vitro* Assays

#### 2.2.1. DPPH Scavenging Activity

**Figure 3:**
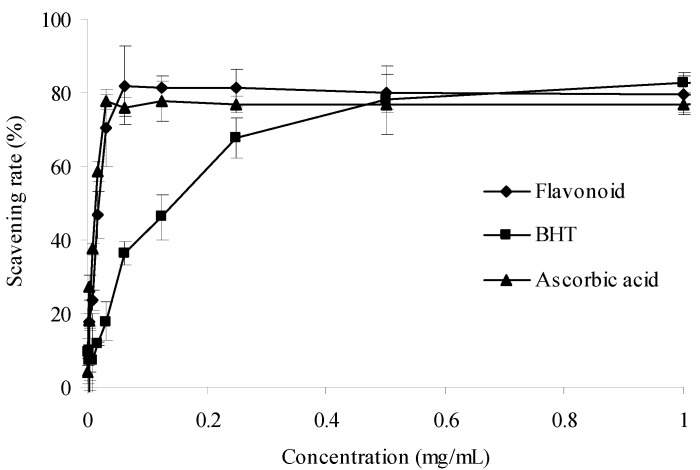
DPPH radical scavenging activities of sample and control standards. Each value is expressed as a mean ± S.D (n = 3).

The α,α-diphenyl-β-picrylhydrazyl (DPPH) radical is a stable N-centered radical at room temperature that is widely employed to assess the radical-scavenging properties of antioxidants [23]. A lower absorbance of the reaction mixture indicates a higher DPPH radical-scavenging activity. In Figure 3, the purified flavonoid exhibited a steady increase in scavenging DPPH free radical with the concentration increase. The scavenging ability of purified flavonoid was comparable to that of ascorbic acid, superior to butylated hydroxytoluene (BHT) at less than 500 ìg/mL. Therefore, the purified flavonoids have significant DPPH radical scavenging activity.

#### 2.2.2. ABTS Radical Scavenging Activity

**Figure 4:**
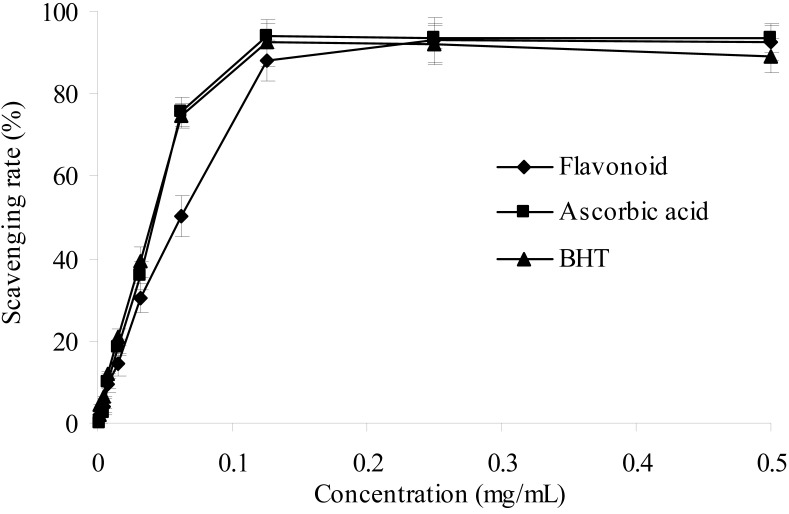
ABTS radical scavenging activities of sample and control standards, Each value is expressed as a mean ± S.D (n = 3).

The 2,2′-azinobis(3-ethylbenzthiazoline-6-sulphonic acid (ABTS) radical cation scavenging assay, which employs a specific absorbance (734 nm) at a wavelength well separated from the visible region and requires a short reaction time, has been extensively applied to evaluate the total antioxidant activity in both lipophilic and hydrophilic samples [24]. As shown in Figure 4, it can be observed to be very effective in scavenging ABTS^+^ radical and the increase was concentration-dependent. Apparently, the purified flavonoid exhibited strongly ABTS^+^ radical scavenging effect. When compared to ascorbic acid and BHT, the ABTS^+^ scavenging ability of the purified flavonoid was comparable at high concentration.

#### 2.2.3. Reducing Power

**Figure 5:**
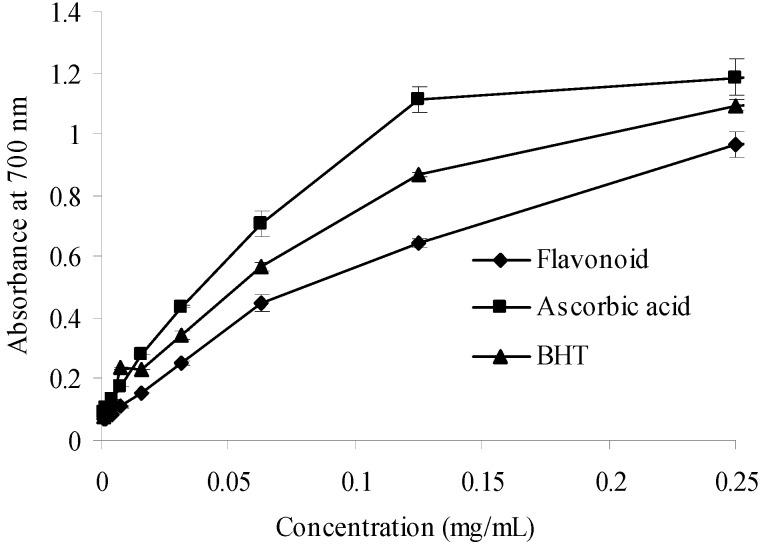
Reducing power of sample and control standards. Each value is expressed as a mean ± S.D (n = 3).

Reducing power is one mechanism of action of antioxidants and may serve as a significant indicator of potential antioxidant activity [25] For the reducing power assay, therefore, the presence of the antioxidant results in the reduction of the Fe^3+^/ferricyanide complex to the Fe^2+^ form. The amount of Fe^2+^ was then monitored by measuring the formation of Perl’s Prussian blue at 700 nm [26]. As shown in Figure 5, a higher absorbance value indicates a stronger reducing power. The purified flavonoid displayed concentration-dependent reducing power. Its reducing power was weaker than those of BHT and ascorbic acid. However, the purified flavonoid exhibited notable antioxidant activity.

#### 2.2.4. Hydroxyl Radical Scavenging Activity

The hydroxyl radicals are very strongly reactive oxygen species formed in biological systems and have been found to damage cellular components of DNA, proteins and lipids, resulting in many health problems such as cancer, aging, and cardiovascular disease [27,28].

**Figure 6:**
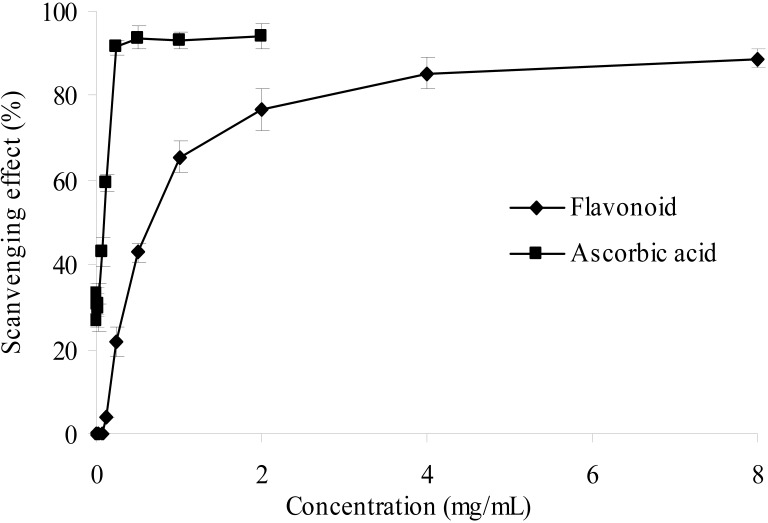
Hydroxyl radical scavenging activities of sample and control standards, Each value is expressed as a mean ± S.D (n = 3).

In this present study, the hydroxyl radical generated Fenton reagent was used to evaluate the scavenging activity of the purified flavonoid. In Figure 6, the results exhibited antioxidant activity in a dose dependent manner, which suggested that the purified flavonoid may help prevent oxidative damage in the human body. The scavenging effect on hydroxyl radical, however, is weaker than that of ascorbic acid.

#### 2.2.5. Superoxide Anion Radical Scavenging Activity

**Figure 7:**
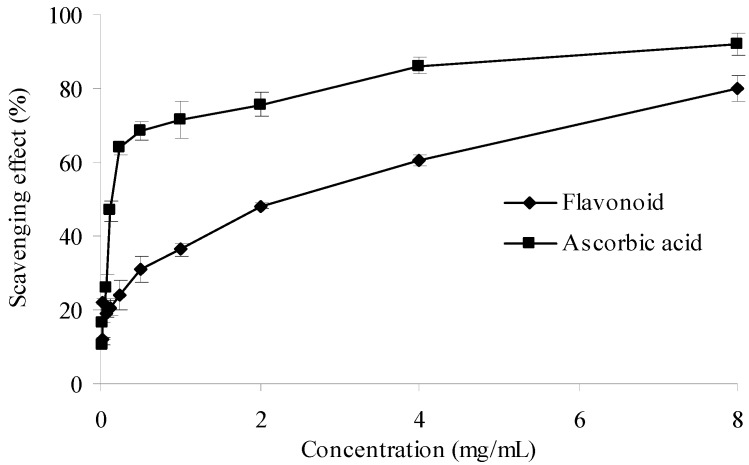
Superoxide anion scavenging activities of sample and control standards. Each value is expressed as a mean ± S.D (n = 3).

Superoxide anion radical is known as an initial radical and considered to play an important role in the peroxidation of lipids [29,30]. The results of superoxide anion scavenging effect of ascorbic acid and the purified flavonoid were given in Figure 7. As illustrated in the figure, both samples showed obvious scavenging activity in a concentration dependent manner. Therefore, the purified flavonoid can be used to scavenge superoxide anion. However, when compared to ascorbic acid, the superoxide scavenging ability of the purified flavonoid at the same concentration was weaker.

## 3. Experimental

### 3.1. Materials and Apparatus

The *Sparganium stoloniferum* Buch.-Ham. was collected from the Dongyang City of Zhejiang Province, China in October, 2010 (N: 29°17′09.76′′, E: 120°19′55.32′′) and authenticated by the corresponding author. The material was allowed to dry naturally and cut into slices, and then ground to pass through a 60-mesh sieve. The powder was kept in sealed polyethylene bags at 4 °C until use. Rutin, kaempferol and formononetin were purchased from the National Institute for the Control of Pharmaceutical and Biological Products (Beijing, China). DPPH, BHT and ABTS were purchased from Sigma-Aldrich (St. Louis, MO, USA). Assay kits for superoxide anion radical (*O_2_*^−^) and hydroxyl radical (OH·) were purchased from Nanjing Jiancheng Bioengineering Institute (Nanjing, China). All other chemical reagents used in experiments were of analytical grade and doubly distilled water was used throughout the experiments. An ultrasonic device (KH300SP, 25 kHz, 300 W, Kunshan Ultrasonic Instrument Co. Jiangsu, China) equipped with a digital timer and a temperature controller was used for ultrasonic extraction of the flavonoids, and a 2501 UV-Vis spectrometer (Shimadzu, Kyoto, Japan) was used for the flavonoids analysis of samples.

### 3.2. Ultrasound-Assisted Extraction of Flavonoids

Two grams of SR powder were placed in a capped tube and mixed with ethanol. The extraction process was carried out using a ultrasonic device. After ultrasonic extraction, the sample was centrifuged at 4,000 rpm for 15 min to collect the supernatant. After being diluted with the extraction solution, UV-Vis analyses were performed. The ethanol concentration, extraction time and ultrasonic power were assessed as shown in the results.

### 3.3. Experimental Design

Box–Behnken Design (BBD), a widely used form of RSM, was selected for the optimisation of the ultrasonic-assisted extraction process and a three-variable, three-level BBD was used. The main factors affecting extraction efficiency, including the ethanol concentration (%, *X_1_*), the extraction time (min, *X_2_*) and the ultrasound power (W, *X_3_*), were selected as independent variables that should be optimised for the extraction of total flavonoids. The temperature was not considered in the present work because the sample was kept at room temperature to avoid the degradation of temperature-sensitive compounds. Although it is generally believed that the usage of larger volume of extracting solvent could obtain larger amount of extract [31], there was the maximum yield at ratio solvent-to-material of 20 mL/g [32]. Therefore, 20 mL/g was set in our study and not investigated further. The independent variables and their levels for the BBD are shown in Table 3. The complete design was carried out in random order and consisted of 17 combinations including five replicates at the central point (Table 1). The data from BBD were analysed by multiple regression to fit the above mentioned quadratic polynomial model (Equation 1).

### 3.4. Conventional Extraction

Conventional extraction was performed according to the method of Mao *et al*. [33] with some modifications. Briefly, 5 grams of SR powder were mixed with 50% ethanol concentration of 100 mL in a 250 mL round-bottom flask. The temperature was maintained at 80 °C for 4 h. After extraction, the sample was centrifuged at 4,000 rpm for 15 min to collect the supernatant. After being diluted with the extraction solution, UV-Vis analyses were performed.

### 3.5. Purification of Flavonoids by Polyamide Resin

The crude extract of flavonoids obtained under the optimized condition was purified using a column packed with polyamide resin. The 95% (v/v) ethanol was used for desorption solvent. The purified extract of flavonoids was collected, and the solvent was evaporated by rotary evaporation at 35 °C. The residue was lyophilized and the resulting dry powder was stored at 4 °C before usage.

### 3.6. Determination of Total Flavonoids

Total flavonoids content was measured using a previously described method [34] with some modification. Briefly diluted sample (1 mL) was placed in a 10 mL volumetric flask. Then NaNO_2_ (0.5 mL, 5%) was added. Next, AlCl_3_ (0.5 mL, 10%) was added 6 min later. After 6 min, 1 M NaOH (4 mL) was added. Distilled water was added to form a 10 mL solution. The solution was mixed thoroughly and the absorbance was measured against a blank at 510 nm. Rutin was used as the standard for a calibration curve.

### 3.7. HPLC Analysis of Flavonoids

HPLC analysis were carried out on a Waters 2,695 chromatograph using a photodiode array detector and a C_18_ reversed-phase column (SunFire^TM^; 5.0 ìm, 150 mm × 4.6 mm; Waters), operated at 30 °C. The mobile phase consisted of a gradient elution of methanol (solvent A) and 0.2% acetic acid (solvent B). The gradient program was: 0-10 min from 30 to 40% of A, 10–20 min 40–55% of A, 20–30 min 55–60% of A, 30–40 min 60–30% A at 1 mL/min. The injected volume was 10 ìL and detection wavelength was 280 nm. Flavonoids were identified by comparing relative retention time and UV-Vis spectra with those of reference standards.

### 3.8. Evaluation of Flavonoids Antioxidant Activities *in Vitro*

#### 3.8.1. DPPH Radical Scavenging Assay

A slight modification was made to the method used by Shimada *et al.* [35] to measure the scavenging activity of DPPH free radicals. One milliliter of 0.2 mM DPPH solution in ethanol and purified flavonoid at various concentrations in 50% ethanol (1 mL) were mixed. The mixture was shaken vigorously and allowed to reach a steady state at room temperature for 45 min. The absorbance of solution was measured at 517 nm using a spectrophotometer. BHT and ascorbic acid were used as references. The DPPH radical scavenging activity was calculated according to the following equation:


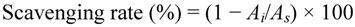



where *A_s_* is the absorbance of pure DPPH, *A_i_* is the absorbance of DPPH in the presence of sample. 

#### 3.8.2. ABTS Radical Scavenging Assay

The ABTS radical cation (ABTS^+^) was employed to detect the antioxidant ability of purified flavonoid according to Re *et al.* [36]. The ABTS is a colorless dianion salt of sodium and can form a colorful ABTS^+^ under oxidation by potassium persulphate. Briefly, ABTS solution (5 mL, 7 mM) and potassium persulphate (5 mL, 2.45 mM) were mixed, and the reaction mixture was left to stand at room temperature in the dark for 16 h. It was then diluted with ethanol to obtain an absorbance of 0.70 ± 0.02 at 734 nm. The ABTS^+^ solution (3.6 mL) was thoroughly mixed with 0.4 mL of the test sample. Various concentrations of test sample (0.4 mL) were incubated with the ABTS^+^ solution for 30 min and the absorbance was recorded at 734 nm against a blank. BHT and ascorbic acid with the same concentrations were used as references. The level of radical scavenging was calculated according to the following equation:


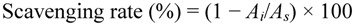


where *A_s_* is the absorbance of pure ABTS^+^, *A_i_* is the absorbance of ABTS^+^ in the presence of sample. 

#### 3.8.3. Reducing Power Assay

The Fe^3+^ reducing power of the sample was measured according to the method of Oyaizu with slight modifications [37]. At various concentrations, flavonoid (1 mL) was mixed with phosphate buffer (2.5 mL, 0.2 M, pH 6.6) and potassium ferricyanide (2.5 mL, 1%, w/v). The mixture was incubated for 20 min at 50 °C. After incubation, TCA (1 mL, 10%, w/v) was added, followed by centrifugation at 3,000 rpm for 10 min. Next, the supernatant (2.5 mL) was mixed with distilled water (2.5 mL) and ferric chloride (FeCl_3_) solution (0.5 mL, 0.1%, w/v) for 10 min. The absorbance at 700 nm was measured as the reducing power. Increased absorbance of the reaction mixture indicated the increased reducing power of the sample. BHT and ascorbic acid were used as the positive controls.

#### 3.8.4. Hydroxyl Radical Scavenging Assay

The hydroxyl radical scavenging kit was used in this assay. The hydroxyl radical wasgenerated from the decomposition of H_2_O_2_ catalyzed by Fe^2+^*in vitro*. When the Griess’ reagent was added, the reaction system produced red substance, which is measured at 550 nm against a blank. Scavenging activity of hydroxyl radical can be assayed by the color change of reaction system. Ascorbic acid was used as positive control. The capability of hydroxyl radical scavenging was calculated as follows:


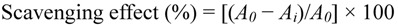


where *A_0_* is the absorbance without sample and *A_i_* is the absorbance with sample.

#### 3.8.5. Superoxide Anion Radical Scavenging Assay

The superoxide anion radical-scavenging kit was used in this assay. In this method, *O_2_^−^* generated *in vitro*, by xanthine-xanthine oxidase system, oxidized hydroxylamine to nitrite in phosphate buffer. Next the Griess’ reagent is added, the reaction system produces red substance, which is measured at 550 nm against a blank. Ascorbic acid was used as positive control. The superoxide radical scavenging ability was calculated as follows:


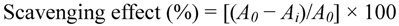


where *A_0_* is the absorbance without sample and *A_i_* is the absorbance with sample.

### 3.9. Statistical Analysis

All the experiments were performed in triplicate in our study. Statistical analyses (*p* < 0.05) were performed using SPSS for Windows, Version 18.0 (SPSS Institute, Inc., Cary, NC, USA), and the regression analysis and the graphical optimization were performed with Design-Expert 8.0 (Trial Version, State-Ease Inc., Minneapolis, MN, USA) software.

## 4. Conclusion

In this study, RSM was successfully employed to optimise the ultrasonic-assistant extraction parameters of the flavonoids from SR. The optimum extraction conditions were obtained and the experimental values for yield of the flavonoids were in close agreement with the predicted ones. The results indicate that ultrasound-assisted extraction is an effective method for the extraction of flavonoids from Sparganii rhizoma. The extraction yields of ultrasound-assisted extraction were higher than those of conventional extraction. The antioxidant activities of the flavonoids *in vitro* including DPPH radical scavenging activity, ABTS^+^ radical scavenging activity, reducing power, hydroxyl radical scavenging activity and superoxide anion scavenging activity, were evaluated, which suggested that the flavonoids showed significant antioxidant activities. The extract was analysed by HPLC and rutin, kaempferol and formononetin were identified. In the future, we will expand the study on the extract of SR to analyse correlation of antioxidant activity and single constituent.
